# Parasitism induces negative effects of clonal integration in the invasive plant *Sphagneticola trilobata* regardless of nutrient heterogeneity

**DOI:** 10.3389/fpls.2026.1947645

**Published:** 2026-10-06

**Authors:** Tian-Xiu Wu, Sai Zhang, Jiangbao Xia, Fang-Lei Gao

**Affiliations:** 1 Shandong Key Laboratory of Eco-Environmental Science for the Yellow River Delta, Shandong University of Aeronautics, Binzhou, China; 2 Tianjin Key Laboratory of Animal and Plant Resistance, College of Life Sciences, Tianjin Normal University, Tianjin, China

**Keywords:** clonal integration, *Cuscuta chinensis*, nutrient heterogeneity, parasitism, *Sphagneticola trilobata*

## Abstract

**Introduction:**

Clonal integration typically enhances the fitness of clonal plants in heterogeneous resource environments, whereas it reduces fitness under parasitic stress. However, it remains unclear whether parasitic stress interacts with nutrient heterogeneity to affect the ecological effects of clonal integration.

**Methods:**

We conducted a greenhouse pot experiment using the invasive clonal plant *Sphagneticola trilobata*, with or without one ramet parasitized by *Cuscuta chinensis*, and with or without severing the connection (allowing or preventing integration), under heterogeneous or homogeneous nutrient supply, combined with ^15^N tracing.

**Results and discussion:**

Our results showed that (1) compared with the severed treatment, the connection treatment significantly increased *C. chinensis* biomass and markedly decreased the biomass of connected but unparasitized ramets, but did not affect the biomass of directly parasitized ramets, ultimately leading to a significant decrease in the total biomass of clonal fragments of *S. trilobata*; (2) heterogeneous nutrient supply significantly intensified the detrimental effect of *C. chinensis* on parasitized ramets, but did not significantly affect connected unparasitized ramets; and (3) neither nutrient heterogeneity nor parasitism by *C. chinensis* affected the amount of ^15^N translocated among ramets. Collectively, these findings indicate that under parasitic stress, clonal integration has detrimental effects on plant fitness. Nutrient heterogeneity intensified the negative effects on parasitized ramets in poor patches but did not alter the detrimental effects on whole clonal fragments. Thus, *C. chinensis* suppresses *S. trilobata* consistently across nutrient conditions, supporting its potential as a biocontrol agent.

## 1 Introduction

Clonal integration refers to the physiological process by which connected ramets within the same clone translocate and share photosynthates, water, mineral nutrients, hormones, and other substances as well as signals (de Kroon and van Groenendael, 1997). The underlying mechanism is widely attributed to the source–sink relationships among connected ramets: when connected ramets differ in resource acquisition capacity or when some ramets are subjected to environmental stress, source–sink gradients are established within the clonal network, driving the translocation and redistribution of resources along these gradients (Alpert, 1991; Wijesinghe and Hutchings, 1999; Hutchings and Wijesinghe, 2008; Dong et al., 2015). A substantial body of empirical evidence has demonstrated that clonal integration can significantly enhance the survival, growth and reproduction of recipient ramets experiencing various environmental stresses, including burial, heavy metal contamination, salinity, herbivory, and competition, thereby effectively increasing the fitness of the clone (Yu et al., 2004; Luo et al., 2017; Portela et al., 2019; Wang et al., 2021b; Guan et al., 2023; Evans et al., 2023; Xu et al., 2025; Li et al., 2026). The general role of clonal integration in mediating the adaptation of clonal plants to environmental stress has been further substantiated by two meta-analyses (Song et al., 2013; Wang et al., 2021a). Thus, clonal integration constitutes a critical ecological strategy for clonal plants to cope with environmental selection pressures (Hutchings, 1999).

However, this general view has been challenged. Recent accumulating experimental evidence indicates that, in the context of plant parasitism, the effects of clonal integration shift from facilitation to suppression, thereby reducing clonal plant fitness (Demey et al., 2015; Těšitelová et al., 2024). In a field removal experiment involving parasitic plants, Demey et al. (2015) found that the abundance of herbaceous species with persistent clonal traits significantly increased after the removal of hemiparasitic herbs from grassland communities, suggesting that clonal plants experience more pronounced growth suppression when subjected to parasitism (Demey et al., 2015). On this basis, Demey et al. (2015) proposed the hypothesis of “parasitism-induced negative effects of clonal integration,” positing that parasitic plants not only directly inhibit the growth of parasitized ramets but also acquire resources from the interconnected unparasitized ramet network via clonal integration, thereby suppressing the growth of those unparasitized ramets and ultimately reducing the fitness of the whole clone. Lepš and Těšitel (2015) further suggested that this process could generate a positive feedback loop: unparasitized connected ramets transfer resources to parasitic plants through clonal integration, which promotes parasitic plant growth, and the vigorous growth of parasitic plants in turn increases their resource demand from the host, thereby exacerbating the suppression of the whole clonal fitness (Lepš and Těšitel, 2015).

The inhibitory effect of clonal integration induced by plant parasitism is fundamentally attributable to resource competition exerted by parasitic plants on their hosts. Since parasitic plants obtain all or part of the substances required for their growth and development from host plants, they act as strong resource sinks (Press et al., 1991; Press and Phoenix, 2005). Upon successful parasitism, a pronounced source–sink relationship is established between the parasite and the host, following which assimilates produced by host photosynthesis are continuously transported to the parasite along the concentration gradient between source and sink tissues (Jeschke et al., 1994; Jeschke and Hilpert, 1997). When a parasitic plant infects a particular clonal ramet, it becomes a superordinate resource sink for the entire clonal network. In this context, resources flow not only directly from the infected ramet to the parasite, but also from the interconnected uninfected ramets through the infected ramet to the parasite via clonal integration. Using *Sphagneticola* species in controlled experiments, Gao et al. (2021a) provided the first direct evidence supporting the above hypothesis: clonal integration significantly promoted the growth of *Cuscuta australis*, did not affect the growth of parasitized ramets, but significantly reduced the growth of connected unparasitized older ramets and the clone. However, this effect was observed only in *Sphagneticola chinensis*, but not in *Sphagneticola trilobata*, indicating that the occurrence of this effect is host-dependent (Gao et al., 2021a). Therefore, whether the negative effects of clonal integration under parasitic stress are generalizable still requires further experimental testing.

In addition to biotic stress such as parasitism, heterogeneous distribution of resources such as light, water, and nutrients represents another critical environmental selection pressure faced by clonal plants. Numerous studies have shown that ramets located in resource-rich patches can transfer resources through clonal integration to ramets in resource-poor patches, significantly enhancing the fitness of the latter without reducing their own fitness, thereby increasing the fitness of the whole clone and effectively alleviating the selection pressure imposed by resource heterogeneity (Xu et al., 2010; Liang et al., 2020; Wang et al., 2017b; Zhang et al., 2022a; Tian et al., 2023). Theoretically, it has been predicted that differences in external resource availability in heterogeneous environments would act in concert with the pre-existing source–sink gradients to modulate the intensity of clonal integration (Wijesinghe and Hutchings, 1999). However, this theoretical prediction has not yet been empirically tested in the context of parasitism. Drawing upon source–sink theory, we further hypothesize that when an infected ramet is situated in a low-nutrient patch while its connected uninfected ramets are located in high-nutrient patches, the external resource disparity would be superimposed upon the source–sink gradient established by parasitism. This would lead to an intensification of clonal integration, enabling the parasitic plant to acquire more resources and consequently exacerbating its detrimental effects on the clonal host plant.

*Sphagneticola trilobata* is a noxious invasive clonal weed in South China, while *Cuscuta chinensis* is a common holoparasitic plant and a well-known traditional Chinese medicine. To test the above hypotheses, we conducted a fully factorial experiment with these two species, involving three two-level factors: presence or absence of *C. chinensis* parasitism, connection or severance of inter-ramet connections, and homogeneous or heterogeneous nutrient supply. The aim was to systematically investigate the effects of *C. chinensis* parasitism, clonal integration, and nutrient heterogeneity on the growth of younger ramets, older ramets, and the whole clonal fragments of *S. trilobata*. Specifically, we tested the following specific hypotheses: (1) clonal integration significantly increases the growth of the parasitic plant *C. chinensis*, has no significant effect on directly parasitized ramets of *S. trilobata*, but significantly suppresses the growth of connected unparasitized ramets, ultimately reducing the total biomass of the whole clonal fragment; and (2) In heterogeneous nutrient conditions, clonal integration significantly promotes the growth of *C. chinensis*, further exacerbating the detrimental effects on connected unparasitized ramets of *S. trilobata*.

## 2 Materials and methods

### 2.1 Study species and material preparation

*Sphagneticola trilobata* (L.) is a perennial clonal herb in the family Asteraceae, genus *Sphagneticola*. Native to tropical regions of South and Central America, this species exhibits strong environmental adaptability, rapid growth, and high dispersal ability, and has caused ecological damage in many tropical and subtropical countries and regions. It has been listed as one of the 100 most invasive alien species worldwide (Lowe et al., 2004). In the 1980s, *S. trilobata* was introduced to Hong Kong, China, as a ground-cover ornamental plant, and subsequently spread rapidly throughout South China. Currently, it has invaded eastern and southern China, as well as coastal areas and islands, primarily inhabiting roadsides, field margins, moist grasslands, and urban green spaces, and has gradually become a noxious invasive weed posing serious threats to agriculture, forestry, and horticulture (Wu et al., 2005).

*Cuscuta chinensis* is an annual holoparasitic herbaceous plant belonging to the genus *Cuscuta* in the family Convolvulaceae. Its stems are slender, filiform, and orange-yellow, lacking roots, with leaves reduced to scales. The flowers are clustered into globular units, with white corollas. The capsules are subglobose, containing 2–4 light brown seeds. The flowering and fruiting period extends from July to October. This plant typically grows on slopes, roadsides, and riverbanks. It obtains nutrients by parasitizing host plants from the Fabaceae, Asteraceae, and Polygonaceae families via haustoria. It is mainly distributed in northern, eastern, central, and southwestern China.

The *S. trilobata* plants used in this study obtained from the laboratory of Professor Yong-Jian Wang at Huazhong Agricultural University. They were collected from five populations in Guangzhou, Guangdong Province, with a minimum distance of 500 m between any two populations to cover different genotypes as much as possible (van Kleunen et al., 2000). The five populations were mixed and cultivated in the research greenhouse of Huazhong Agricultural University. Before the experiment began, the plants were transferred to the greenhouse of Shandong University of Aeronautics and cultivated for 10 months, allowing the production of sufficient numbers of clonal ramet pairs through clonal growth. On 24 July 2021, ramet pairs of similar size were selected and planted in plastic trays (54.5 × 28.5 × 5 cm, length × width × height) filled with peat substrate (Recipe413, Klasmann-Deilmann Produktionsgesellschaft Nord GmbH & Co., Saterland, Germany). All trays were placed in the greenhouse of Shandong University of Aeronautics and irrigated with sufficient water for rooting and establishment.

### 2.2 Experimental design

The experiment used a fully factorial design that crossed two nutrient supply treatments (homogeneity or heterogeneity) with two parasitism treatments (present or absent) with two severance treatments (severed or left connected), with six replicates per treatment. For the severed treatment, the stem was cut halfway between the ramets. For the connected treatment, the stem was left intact. For the treatment with parasitism present, the younger, distal ramet in a pair was infested with *C. chinensis*. Nutrient supply was designed as two treatments: homogeneity and heterogeneity. The total amount of fertilizer applied per clonal fragment over the entire experimental period was identical in both treatments (0.25 g of water-soluble fertilizer, Peters Professional 20-20-20+TE General Purpose Fertilizer, N: P_2_O_5_: K_2_O = 20: 20: 20). For the homogeneous treatment, each ramet (both younger and older) received 0.125 g of fertilizer. For the heterogeneous treatment, the younger ramet received 0.05 g, while the older ramet received 0.20 g. On 8 August 2021, 48 rooted pairs of *S. trilobata* ramets with similar size were selected from the cultivated plants of *S. trilobata*. The fresh weight of each ramet pair was weighed and recorded before planting. Each ramet pair was planted into a pair of plastic pots (18 cm top diameter, 16 cm height, 12 cm bottom diameter), with each pot containing 2 L of artificially prepared growth substrate. The growth substrate consisted of peat (Recipe 413, Klasmann-Deilmann Produktionsgesellschaft Nord GmbH & Co., Saterland, Germany) and vermiculite (Particle size ranging from 0 mm to 3 mm) mixed in equal volumes. All pots were randomly placed on benches in the greenhouse of Shandong University of Aeronautics. Each pot received 50 mL of water-soluble nutrient solution (Peters Professional 20-20-20+TE General Purpose Fertilizer, N: P_2_O_5_: K_2_O = 20: 20: 20) at a concentration of 2 g/L as basal fertilizer after finished planting. The seedlings were allowed to recovery from transplanting for one week, during which dead ramets were replaced promptly.

On 15 August 2021, 24 of the 48 ramet pairs were randomly selected for parasitism treatment on the younger ramet within each pair. *C. chinensis* seedlings of 2–5 cm in length were placed around the younger ramets to facilitate parasitism, with 30 seedlings added per pot to maximize the likelihood of successful parasitism. Parasitism status was monitored continuously; successful establishment was confirmed upon formation of haustoria and emergence of new shoots from the parasite. For treatments in which parasitism was unsuccessful, additional seedlings of *C. chinensis* were supplied until all replicates were successfully parasitized.

On 29 August 2021, 24 randomly selected ramet pairs were subjected to severance treatment, in which the connecting spacers between the paired ramets were cut with scissors; the remaining 24 pairs were left intact. Concurrently, soil nutrient treatments were initiated: 24 pairs were assigned to the homogeneous nutrient treatment, with each pot receiving 5 mL water-soluble nutrient solution (Peters Professional 20-20-20+TE General Purpose Fertilizer, N: P_2_O_5_: K_2_O = 20: 20: 20) at a concentration of 5 g/L. The other 24 pairs received the heterogeneous nutrient treatment, in which older ramet pots received 8 mL and younger ramet pots received 2 mL of the same 5 g/L solution, ensuring that each pot pair received a total of 10 mL of the water-soluble nutrient solution. The treatments lasted for five weeks. During the experimental period, water was supplied as needed to keep the substrate surface moist. The entire experiment was conducted in the research greenhouse at Shandong University of Aeronautics, with a light transmittance of 70.00%, and average temperature and relative humidity of 26.13 °C and 77.19%, respectively, over the experimental period.

### 2.3 Harvest and measurements

On 7 October 2021, four replicates out of six were randomly selected from each combination of nutrient treatment (homogeneous vs. heterogeneous) × parasitism treatment (with vs. without *C. chinensis*) under the connection treatment for ^15^N isotope labeling, with the remaining two replicate serving as the control. To avoid nutrient effects associated with ^15^N labeling, the labeled compound was added at a rate of 1 μg N per gram of growth substrate, resulting in a total addition of 1.5 mg ^15^N-labeled KNO_3_ (96.16 atom%, Shanghai Research Institute of Chemical Industry) per pot pair, all of which was applied to the pot containing the older ramet. The labeled KNO_3_ was dissolved in water and injected using a syringe at three equally spaced points forming a triangle around the *S. trilobata* ramet, with 10 mL of solution injected at each point. Two hours after labeling, the aboveground biomass of younger and older ramets of *S. trilobata*, as well as biomass of *C. chinensis*, was harvested. Belowground biomass of both younger and older ramets was collected using the rinsing method. At the end of the experiment, one replicate in the homogeneous nutrient × with parasitism × connection treatment was excluded from subsequent data analysis due to mortality of *C. chinensis*. All harvested plant materials were placed in envelopes and dried in an oven at 70 °C to constant weight, after which dry biomass was recorded. After biomass measurement, the samples were ground to a fine powder using a ball mill (Retsch MM 200, Germany). Approximately 2 mg of each sample was accurately weighed, and the total ^15^N/^14^N ratio was measured using a stable isotope mass spectrometer (Elementar vario ISOTOPE cube, Germany). APE (%) was calculated using Equation 1, and ^15^N uptake (µg) was calculated using Equation 2, following Liu et al. (2025):

(1) $$\text{APE}(\%)={\text{atom\%}}^{15}{\text{N}}_{\text{labeled}}-{\text{atom\%}}^{15}{\text{N}}_{\text{unlabeled}}$$

(2) N15 uptake (μg) = Biomass (g) × APE (%) × N% × 100

where APE is the atom percent excess of ^15^N (%), atom% ^15^N_labeled_ is the ^15^N atom percentage of labeled plants, atom% ^15^N_unlabeled_ is the ^15^N atom percentage of unlabeled plants, Biomass is the plant dry biomass (g), and N% is the plant nitrogen content (%).

### 2.4 Statistical analysis

To examine the effects of nutrient heterogeneity, severance treatment, parasitism treatment, and their interactions on younger ramet biomass (aboveground, belowground, and total), older ramet biomass (aboveground, belowground, and total), and the clonal fragment biomass (combined younger and older ramets) of *S. trilobata*, we employed generalized linear models (GLMs). In the models, the initial fresh weight of each ramet pair was included as a covariate, while nutrient heterogeneity, severance treatment, parasitism treatment, and their interactions were specified as fixed factors. Biomass data were assumed to follow a Gamma distribution with a log-link function. Graphical diagnostic methods, including residual versus fitted value scatterplots and Q-Q plots, were used to examine the residual distribution patterns, ensuring no obvious trends in residuals and confirming the linearity assumption between independent variables and the dependent variable transformed by the link function. The independence and multicollinearity assumptions were tested using the *durbinWatsonTest* and *vif* functions from the car package, respectively. The fixed effects were estimated using the *Anova* function with Type III sums of squares. For cases with significant two-way interactions, simple effect analyses were conducted using the *pairs* function from the *emmeans* package.

For *C. chinensis* biomass, ^15^N uptake by *C. chinensis*, and ^15^N uptake by aboveground tissues of *S. trilobata* younger and older ramets, GLMs were similarly applied. The initial fresh weight of the *S. trilobata* ramet pair was included as a covariate and nutrient treatment, parasitism treatment, and their interactions were set as fixed factors. The data were also assumed to follow a Gamma distribution with a log-link function. The same graphical diagnostic methods (residual vs. fitted value scatterplots, Q-Q quantile plots) were employed to examine residual distribution patterns and ensure the absence of obvious trends in residuals, as well as to verify the linearity assumption. The independence and multicollinearity assumptions were tested using the *durbinWatsonTest* and *vif* functions from the car package. The fixed effects were estimated using the *Anova* function with Type III sums of squares. All analyses were performed using R version 4.2.2 (R Core Team, 2022), and figures were created using OriginPro 2025.

## 3 Results

### 3.1 Biomass of *S. trilobata* younger ramets

In the present of *C. chinensis*, nutrient heterogeneity significantly decreased both aboveground biomass (**Figure 1a**; **Supplementary Figure S1a**), and total biomass (**Figure 1c**; **Supplementary Figure S1c**) of younger ramet, but not in the absent of *C. chinensis*, as indicated by the significant interactive effect of nutrient heterogeneity × parasitism by *C. chinensis* (**Table 1**). Clonal integration had no significant effect on younger ramet biomass (**Table 1**). No significant three-way interactive effects among nutrient heterogeneity, parasitism by *C. chinensis* and clonal integration were detected for aboveground, belowground or total biomass of younger ramets (**Table 1**). No significant two-way interactions were found between nutrient heterogeneity and clonal integration, nor between parasitism by *C. chinensis* and clonal integration, for any of the three biomass variables of younger ramets (**Table 1**).

**Figure 1:**
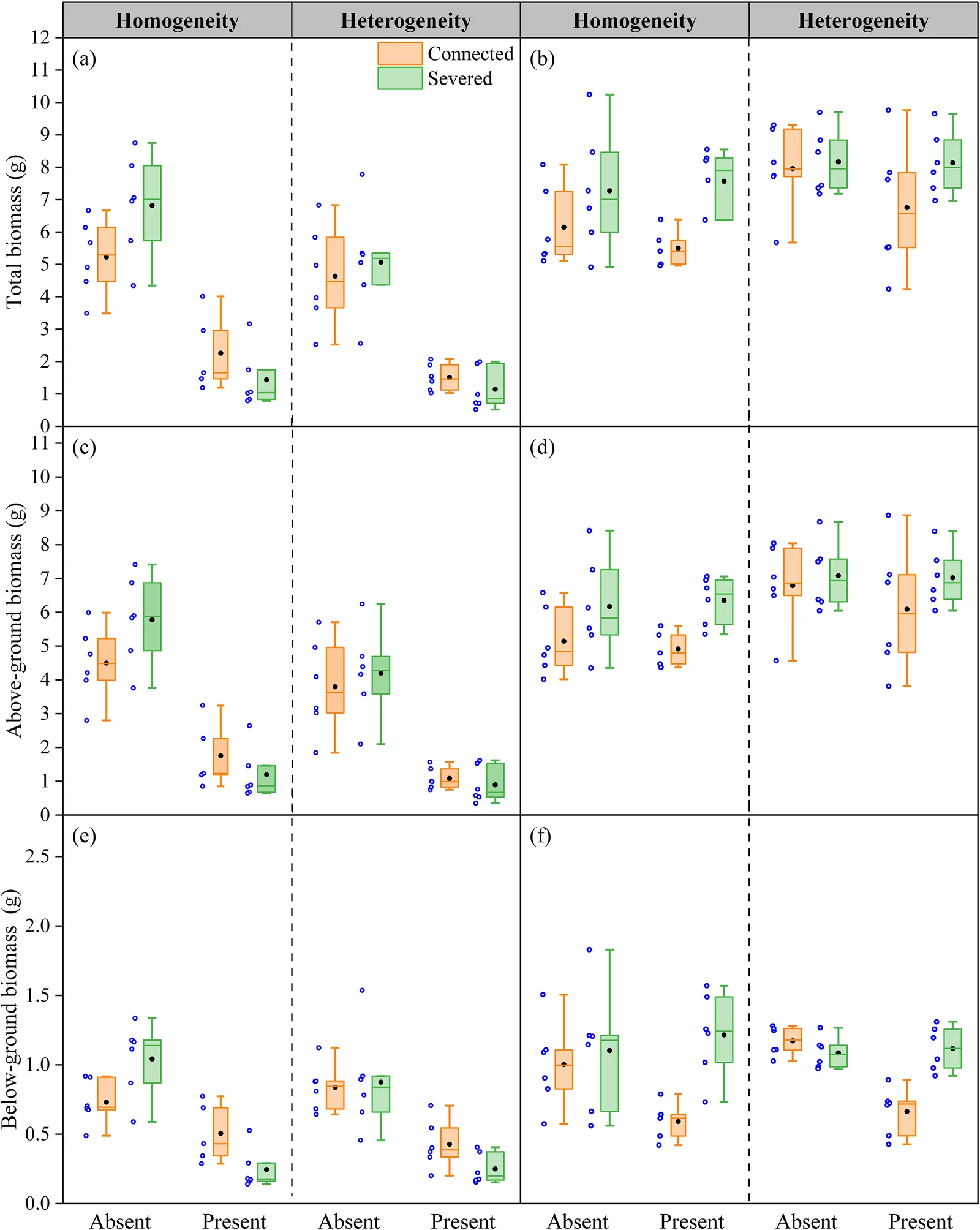
Biomass of *S. trilobata* in response to nutrient heterogeneity, parasitism by *C. chinensis*, and clonal integration. **(a)** Total biomass of younger ramets, **(b)** total biomass of older ramets, **(c)** aboveground biomass of younger ramets, **(d)** aboveground biomass of older ramets, **(e)** belowground biomass of younger ramets, and **(f)** belowground biomass of older ramets.

**Table 1:** Three-way ANOVA Results for the effects of nutrient heterogeneity, parasitism by *C. chinensis*, and severance treatment on the biomass of younger and older ramets.

		Younger ramet						Older ramet					
Effect		Above-ground mass		Below-ground mass		Total mass		Above-ground mass		Below-ground mass		Total mass	
	*df*	χ^2^	*P*	χ^2^	*P*	χ^2^	*P*	χ^2^	*P*	χ^2^	*P*	χ^2^	*P*
Initial fresh mass	1	33.16	**<0.001**	15.77	**<0.001**	30.91	**<0.001**	8.70	**0.003**	9.67	**0.002**	9.98	**0.002**
Heterogeneity (H)	1	20.38	**<0.001**	4.87	**0.027**	16.69	**<0.001**	0.43	0.512	0.26	0.612	0.26	0.614
Parasitism (P)	1	73.53	**<0.001**	24.73	**<0.001**	64.70	**<0.001**	5.67	**0.017**	27.21	**<0.001**	8.93	**0.003**
Severance (S)	1	0.04	0.835	3.29	*0.070*	0.10	0.752	5.32	**0.021**	22.3	**<0.001**	7.96	**0.005**
H × P	1	7.94	**0.005**	4.55	**0.033**	7.01	**0.008**	1.84	0.175	1.45	0.229	1.89	0.170
H × S	1	2.48	0.115	1.72	0.190	2.27	0.132	0.03	0.876	0.10	0.751	0.05	0.816
P × S	1	0.02	0.878	1.17	0.279	0.03	0.855	2.53	0.112	15.60	**<0.001**	4.30	**0.038**
H × P × S	1	2.04	0.153	2.86	*0.091*	2.16	0.142	0.25	0.619	0.05	0.826	0.20	0.656

Values in bold indicate *P*< 0.05, and values in *italics* indicate 0.05≤*P*< 0.1.

### 3.2 Biomass of *S. trilobata* older ramets

Parasitism by *C. chinensis* and clonal integration had a significant interactive effect on belowground (**Figure 1d**) and total biomass (**Figure 1f**) of *S. trilobata* older ramets (**Table 1**). In the absence of parasitism, clonal integration had no significant effect on belowground or total biomass of older ramet (**Supplementary Figures S2a, b**); however, in the presence of parasitism, clonal integration significantly reduced both belowground and total biomass of older ramet (**Supplementary Figures S2a, b**). No significant interactive effect was detected for aboveground biomass of older ramet (**Figure 1b**; **Table 1**). No significant three-way interactions among nutrient heterogeneity, parasitism and clonal integration were found for aboveground, belowground or total biomass of older ramets (**Table 1**). Neither the two-way interaction between nutrient heterogeneity and clonal integration nor that between nutrient heterogeneity and parasitism significantly affected on aboveground, belowground or total biomass of older ramet (**Table 1**). Nutrient heterogeneity alone had no significant effect on aboveground, belowground or total biomass of older ramets (**Table 1**).

### 3.3 Biomass of *S. trilobata* the clonal fragment

Both parasitism by *C. chinensis* and clonal integration significantly reduced the biomass of the whole clonal fragment of *S. trilobata*, and their interaction was marginally significant (*P* = 0.066, **Table 2**; **Figure 2**). To explore this trend, we conducted *post-hoc* comparisons and found that in the presence of parasitism, clonal integration significantly decreased the biomass of the whole clonal fragment, whereas in the absence of parasitism, clonal integration had no significant effect on the whole clonal fragment biomass (**Supplementary Figure S2c**). However, because the interaction was only marginally significant, these *post-hoc* results should be interpreted with caution and warrant further verification with larger sample sizes. Nutrient heterogeneity, as well as all two-way and three-way interactions involving nutrient heterogeneity, were not significant (**Table 2**).

**Table 2:** Three-way ANOVA results for the effects of nutrient heterogeneity, parasitism by *C. chinensis*, and clonal integration on the clonal fragment of *S. trilobata*, and two-way ANOVA results for the effects of nutrient heterogeneity and clonal integration on *C. chinensis* biomass.

Effect		Combined mass of ramets		*C. chinensis* biomass	
	*df*	χ^2^	*P*	χ^2^	*P*
Initial fresh mass	1	20.54	**<0.001**	21.16	**<0.001**
Heterogeneity (H)	1	1.47	0.225	10.12	**0.001**
Parasitism (P)	1	37.82	**<0.001**	—	—
Severance (S)	1	6.93	**0.008**	21.72	**<0.001**
H × P	1	2.83	*0.093*	—	—
H × S	1	0.46	0.499	0.02	0.884
P × S	1	3.39	*0.066*	—	—
H × P × S	1	1.57	0.211	—	—

Values in bold indicate *P*< 0.05, and values in *italics* indicate 0.05≤*P*< 0.1.

**Figure 2:**
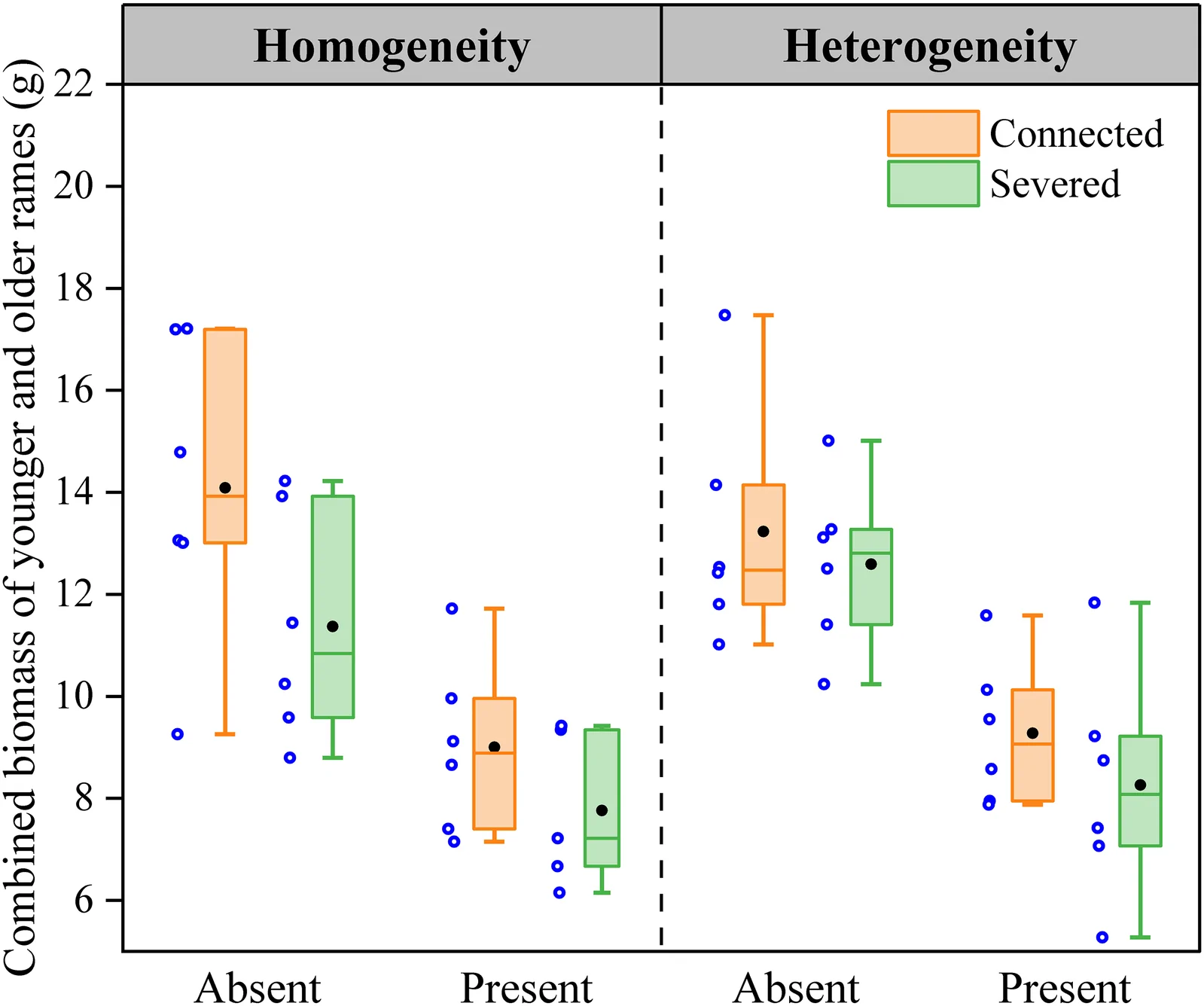
Biomass of the *S. trilobata* clonal fragment in response to nutrient heterogeneity, parasitism by *C. chinensis*, and clonal integration.

### 3.4 Biomass of *C. chinensis*

Compared with homogeneous nutrient supply, heterogeneous nutrient supply significantly reduced the biomass of *C. chinensis* (**Table 2**; **Figure 3a**). Clonal integration significantly increased the biomass of *C. chinensis* compared with the severance treatment. (**Table 2**; **Figure 3a**). However, no significant interaction effect between soil nutrient heterogeneity and clonal integration was detected on the biomass of *C. chinensis* (**Table 2**).

**Figure 3:**
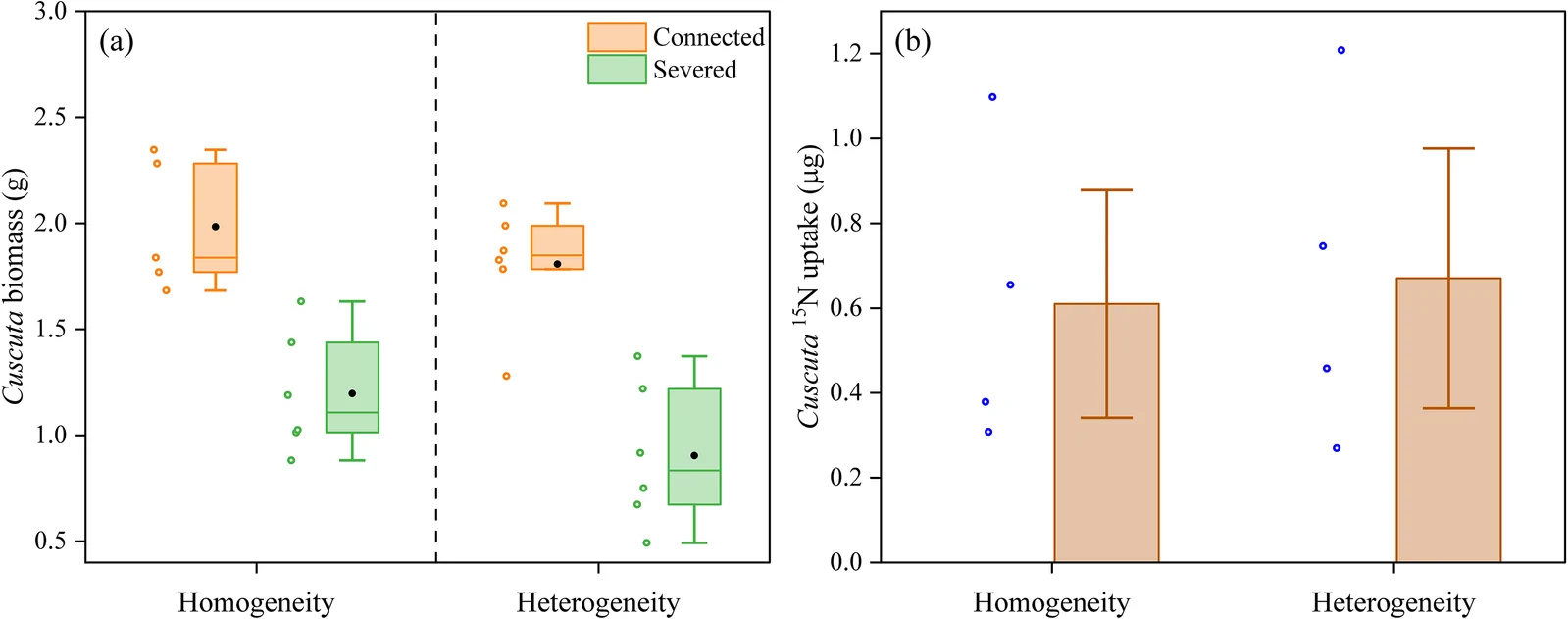
**(a)** *C. chinensis* biomass in response to nutrient heterogeneity and clonal integration, and **(b)** ¹^5^N uptake by *C. chinensis* in response to soil nutrient heterogeneity.

### 3.5 ¹^5^N uptake

Compared with heterogeneous nutrient supply, homogeneous nutrient supply did not significantly affect the ¹^5^N uptake of *C. chinensis* (**Table 3**; **Figure 3b**). Likewise, homogeneous nutrient supply had no significant effect on the aboveground ¹^5^N uptake of either younger or older ramets of *S. trilobata* relative to heterogeneous supply (**Table 3**; **Figures 4a, b**). Clonal integration also did not significantly affect the aboveground ¹^5^N uptake of younger or older ramets of *S. trilobata* compared with the severance treatment (**Table 3**; **Figures 4a, b**). Furthermore, no significant interaction effect between soil nutrient heterogeneity and clonal integration was detected on the aboveground ¹^5^N uptake of either younger or older ramets of *S. trilobata* (**Table 3**; **Figures 4a, b**).

**Table 3:** Two-way ANOVA results for the effects of nutrient heterogeneity and parasitism by *C. chinensis* on aboveground ¹^5^N uptake by older and younger ramets of *S. trilobata*, and one-way ANOVA results for the effect of nutrient heterogeneity on ¹^5^N uptake by *C. chinensis*.

Effect		*C. chinensis* ^15^N uptake		Younger ramet above-ground ^15^N uptake		Older ramet above-ground ^15^N uptake	
	*df*	χ^2^	*P*	χ^2^	*P*	χ^2^	*P*
Initial fresh mass	1	2.840	*0.092*	0.776	0.378	2.690	0.101
Heterogeneity (H)	1	2.292	0.130	0.388	0.533	0.597	0.439
Parasitism (P)	1	—	—	0.143	0.705	0.009	0.925
H × P	1	—	—	3.494	*0.062*	0.182	0.670

Values in *italics* indicate 0.05≤*P*< 0.1.

**Figure 4:**
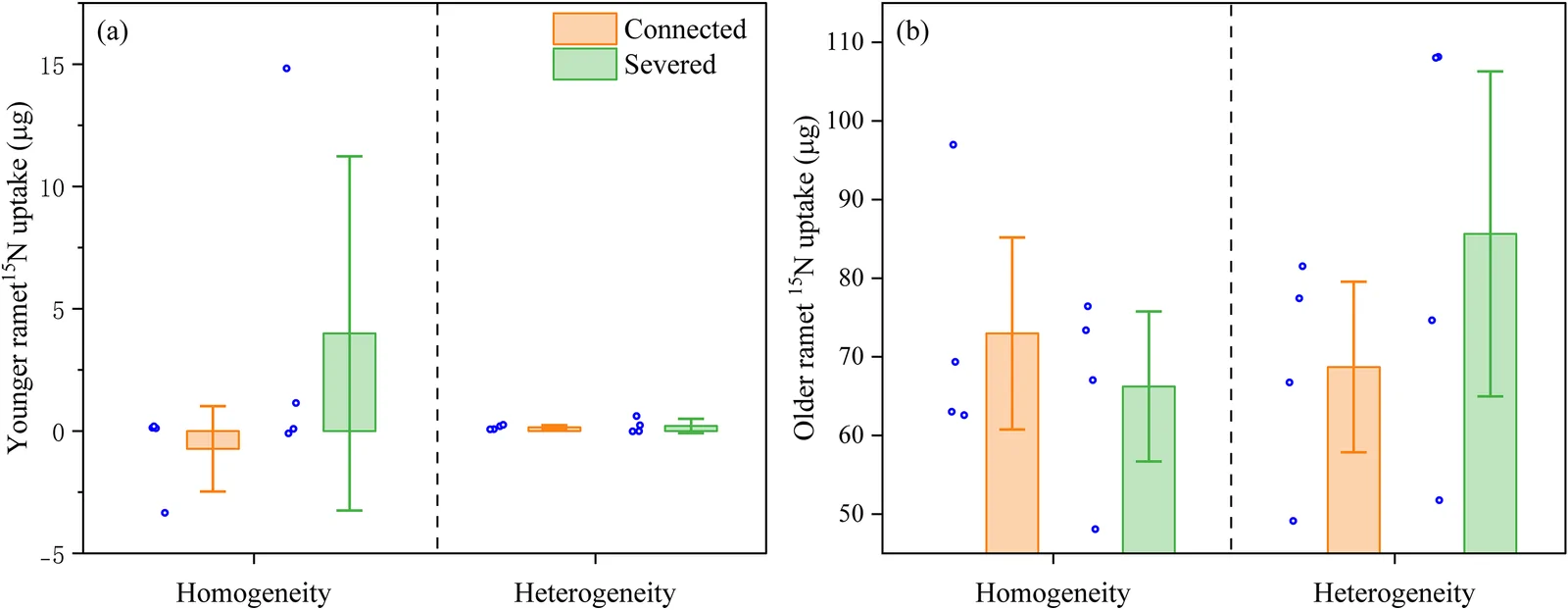
¹^5^N uptake by **(a)** younger and **(b)** older ramets of *S. trilobata* in response to soil nutrient heterogeneity and clonal integration.

## 4 Discussion

The results of the present study support our first hypothesis. In the presence of *C. chinensis* parasitism, clonal integration significantly promoted growth of *C. chinensis* (**Figure 3a**), had no significant effect on the biomass of parasitized ramets of *S. trilobata* (**Table 1**), but significantly reduced the biomass of connected unparasitized ramets (**Supplementary Figure S2b**), thereby decreasing the fitness of the clonal fragment of *S. trilobata* (**Supplementary Figure S2c**). These findings are consistent with the parasitism–clonal integration hypothesis (Demey et al., 2015; Lepš and Těšitel, 2015), indicating that parasitism by *C. chinensis* induces a negative effect of clonal integration on the connected unparasitized ramets of *S. trilobata*.

Our results share both similarities and differences with those of Gao et al. (2021a). In similarity, both studies confirmed that parasitism by the dodder can induce negative effects of clonal integration. The difference lies in the host-dependent nature of this effect reported by Gao et al. (2021a): parasitism by *Cuscuta australis* induced negative effects only on *Sphagneticola chinensis*, but not on *S. trilobata*. To further elucidate this discrepancy, we integrated data from previous studies and the present work, and found that regardless of whether the parasite was *C. australis* or *C. chinensis*, high-intensity parasitism significantly reduced the fitness of connected unparasitized ramets of the host plant (*S. chinensis* or *S. trilobata*), whereas low-intensity *C. australis* parasitism on *S. trilobata* did not produce such an effect (**Supplementary Figure S3**). This cross-study comparison suggests, albeit speculatively, that parasitism intensity might be a potential factor influencing whether the negative effect of clonal integration occurs. However, because our experiment did not directly manipulate parasitism intensity, this inference should be interpreted with caution. The underlying mechanism might be that the dodder acts as a powerful carbon sink (Yuan et al., 2023); higher parasitism intensity entails a greater demand for photosynthates, thereby forcing connected unparasitized ramets to continuously export resources through clonal integration. Sustained high-level resource export ultimately inhibits the growth and biomass accumulation of unparasitized ramets, reducing their fitness and consequently inducing the negative effect of clonal integration (Gao et al., 2021a; Těšitelová et al., 2024). On this basis, we tentatively propose a working hypothesis—the “parasitism intensity–clonal integration hypothesis”—as a direction for future research: whether the negative effect of clonal integration occurs may depend on whether parasitism intensity exceeds a certain threshold. Clearly, this hypothesis requires direct testing through controlled experiments with manipulated parasitism intensities. Future studies are therefore needed to test this hypothesis, ideally through controlled experiments with manipulated parasitism intensities, to determine whether a threshold exists and to quantify it. Such work would also provide a more robust theoretical basis for the biological control of invasive clonal plants using the dodder.

Clonal integration, as an important ecological adaptive strategy of clonal plants, effectively alleviates various environmental stresses—such as sand burial, heavy metal contamination, flooding, and resource patchiness—through resource sharing among interconnected ramets. It has been generally accepted that clonal integration enhances the survival and growth of recipient ramets without significantly compromising the fitness of donor ramets, thereby increasing overall clonal fitness (Song et al., 2013; Liu et al., 2016; Wang et al., 2021a). However, the present study reveals a phenomenon contrary to this consensus: under parasitism stress by the dodder, clonal integration did not significantly reduce the biomass of parasitized ramets (**Table 1**; **Figure 2a**), but significantly suppressed the growth of connected unparasitized ramets (**Supplementary Figure S2b**), ultimately decreasing the fitness of the entire clone. This indicates that, under parasitic stress, clonal integration does not perform its classic adaptive function, but rather shifts to a non-adaptive strategy.

This discrepancy might stem from the unique nature of parasitic stress, which distinguishes it from other environmental stresses. Previous studies have focused on abiotic stresses such as drought and flooding, whose detrimental effects are generally constant over time (Zhang et al., 2022b; Duan et al., 2024; Liu et al., 2024). Under such stresses, unstressed donor ramets can enhance resource uptake and export efficiency through physiological activation without reducing their own fitness (Tian et al., 2023; Zhao et al., 2023). In contrast, biotic stresses such as herbivory and trampling are fluctuating, but clonal plants can buffer these through reciprocal intra-clonal division of labor: aboveground-damaged ramets enhance belowground resource acquisition, while undamaged ramets strengthen photosynthesis and export carbon to damaged ramets, forming a bidirectional beneficial exchange (Stuefer et al., 1996; Wang et al., 2017a; Zhang et al., 2023). Parasitic stress, however, possesses a distinctly different characteristic—its detrimental effects intensify progressively with the growth and development of the parasite (Lepš and Těšitel, 2015). As the dodder biomass increases, its resource demand as a powerful carbon sink continuously escalates (Yuan et al., 2023; Gao et al., 2021a). Moreover, parasitic exploitation not only deprives parasitized ramets of photosynthates, mineral nutrients, and water, but may also disrupt the inherent clonal division-of-labor capacity of the host—for example, by inhibiting root biomass allocation or reducing root uptake efficiency (Cirocco et al., 2017; Gao et al., 2019). In this context, parasitized ramets are unable to effectively compensate for resource losses on their own, and instead continuously rely on resource export from connected unparasitized ramets. As parasitism intensity escalates, resources from unparasitized ramets are unidirectionally drawn out over an extended period, ultimately suppressing their growth and cascadingly reducing the fitness of the entire clone. Therefore, the negative effect of clonal integration induced by parasitic stress fundamentally reflects an ecological process distinct from that of abiotic stresses and fluctuating biotic stresses: the resource demand of the parasite increases in a positive feedback manner over time, transforming clonal integration from a risk-sharing strategy into a risk transfer strategy, ultimately impairing overall clonal fitness.

The results of the present study did not support our second hypothesis; however, a significant interactive effect between nutrient heterogeneity and parasitism on the growth of *S. trilobata* younger ramets was detected. Specifically, parasitism by *C. chinensis* significantly suppressed the growth of younger ramets, and this suppressive effect was more pronounced under heterogeneous nutrient supply (**Supplementary Figure S1**). To explain the underlying causes of this exacerbated effect, we first ruled out the possibility that the lower total nutrient amount per se in the heterogeneous treatment imposed stress on the host plants. Our data showed that, in the absence of parasitism, there was no significant difference in younger ramet biomass between the heterogeneous treatment (150 mL of nutrient solution received by younger ramets) and the homogeneous treatment (225 mL received by younger ramets) (**Supplementary Figure S1c**), indicating that the lower nutrient concentration in the heterogeneous treatment was still sufficient to maintain normal growth of *S. trilobata* younger ramets and did not constitute nutrient limitation. Therefore, the exacerbation of parasitic damage under heterogeneous conditions might be attributed to the interaction between parasitic pressure and nutrient supply, rather than to nutrient stress *per se*.

Further analysis indicated that higher nutrient supply effectively alleviated the detrimental effects of the parasite (Shen et al., 2013; Gao et al., 2021b). Among parasitized ramets, biomass was significantly greater under the homogeneous high-nutrient treatment (225 mL) than under the heterogeneous low-nutrient treatment (150 mL) (**Supplementary Figure S1c**), suggesting that high-nutrient conditions might enhance host tolerance to parasitism. The underlying mechanism may be that parasitism by *C. chinensis* diverts substantial photosynthates that would otherwise be allocated to host growth and development, thereby impairing the construction of resource-acquiring organs such as roots. Under higher nutrient availability, host roots can more readily acquire mineral nutrients and water, partially compensating for the material losses caused by parasitism and mitigating its detrimental effects. This inference is consistent with the findings of Gao et al. (2021b).

Contrary to the hypothesis that heterogeneous nutrient supply promotes growth of the dodder, the present study found that nutrient heterogeneity significantly reduced *C. chinensis* biomass (**Table 2**; **Figure 3a**). The logical basis of this hypothesis was that heterogeneous nutrient conditions would create a larger resource gradient, thereby facilitating resource flow toward the host and cascading enhancing fitness of the dodder; however, our results did not support this expectation. Combined with the preceding analysis, we tentatively infer that the reduced growth of *C. chinensis* under heterogeneous conditions may not be directly attributable to nutrient stress on the host *per se*. In the absence of parasitism, no significant difference in host biomass was observed between the heterogeneous (total 150 mL) and homogeneous (225 mL) treatments, suggesting that the lower nutrient concentration was sufficient to sustain normal host growth. However, in the presence of *C. chinensis*, host biomass was significantly lower under heterogeneous nutrient supply than under homogeneous supply (**Supplementary Figure S1c**). This contrast implies that the reduced growth of *C. chinensis* under heterogeneity may be related to a reduced tolerance of the host to parasitic stress under heterogeneous conditions, which could in turn limit the surplus resources available for exploitation by *C. chinensis*. Clearly, this inference remains speculative and requires direct physiological validation (e.g., measurements of host root uptake efficiency, photosynthetic rate, or phloem loading) in future controlled experiments before it can be established as a mechanistic conclusion.

Severance treatment also significantly reduced the biomass of *C. chinensis* (**Table 2**; **Figure 3a**), which is consistent with our prior expectation. Severance disrupted clonal integration, thereby blocking material translocation from connected unparasitized ramets to the dodder and consequently reducing parasite fitness. This finding is in agreement with the results of Gao et al. (2021a). Furthermore, although nutrient heterogeneity treatment significantly reduced the biomass of *C. chinensis*, the ¹^5^N labeling experiment did not detect significant effects of nutrient heterogeneity on ¹^5^N translocation (**Table 2**; **Figure 3**). This seemingly contradictory result may be attributable to two reasons. First, ¹^5^N labeling reflects the instantaneous or short-term translocation rate of mineral elements, whereas the dodder biomass accumulation results from the net input of materials (particularly photosynthates) over a prolonged period, indicating a mismatch in temporal scales. Second, the relatively large variability within treatments (**Figure 3b**) may have weakened the statistical significance of differences among treatments. We suggest that future studies increase the number of replicates or employ multiple labeling time points (e.g., at the early, middle, and late stages of the experiment) to more comprehensively capture the temporal dynamics of material translocation via clonal integration and to reduce random errors associated with single-time-point labeling.

## 5 Conclusions

The results of the present study demonstrate that, under plant parasitism stress, clonal integration significantly enhances the fitness of the parasitic plant (*C. chinensis*) at the cost of reducing the overall fitness of the clonal genet. This negative effect is primarily achieved through the suppression of growth in unparasitized ramets connected to the parasitized ones, whereas the fitness of the parasitized ramets themselves is not significantly affected. Our results suggest that, under parasitic stress, clonal integration may reverse its beneficial effect on the clonal plant, shifting from positive to negative.

Further analysis indicates that the intensity of this negative effect is independent of resource heterogeneity; however, the presence of parasitic plants exacerbates the decline in fitness of clonal ramets located in low-resource patches, suggesting that clonal populations in resource-poor habitats may face greater threats from parasitism. Given that *Sphagneticola trilobata*, as a globally notorious invasive clonal weed, poses severe threats to ecosystems, and that *C. chenensis*, as an obligate parasitic plant, has been proven to effectively suppress its growth and spread, the present study supports the application of *C. chinensis* as a biological control agent in the practical management of *S. trilobata*, despite the suppressive effect of parasitic stress on overall clonal fitness did not affect by nutrient heterogeneity. In future applications, parasitic intensity, habitat resource conditions, and potential impacts on non-target species should be comprehensively considered to optimize control strategies and minimize ecological risks.

## Data Availability

The original contributions presented in the study are included in the article/**Supplementary Material**. Further inquiries can be directed to the corresponding author.
