# Supplementary material for: Parasitism induces negative effects of clonal integration in the invasive plant Sphagneticola trilobata regardless of nutrient heterogeneity
Source: Front Plant Sci. 2026 Oct 6;17:1947645. doi: 10.3389/fpls.2026.1947645 (PMC13680194; doi:10.3389/fpls.2026.1947645)
Supplement: Supplementary file 1 [file DataSheet1.docx]

**Supplementary Figures**

**Appendix Fig. S1 to S3**

**Fig. S1.** Biomass of *S. trilobata* younger ramets in response to soil nutrient heterogeneity and *Cuscuta* parasitism. Note: (a) belowground biomass, (b) aboveground biomass, and (c) total biomass. Numbers above boxes give probability that means did not differ between parasitism or nutrient treatments.

**Fig. S2.** Biomass of *S. trilobata* in response to *Cuscuta* parasitism and clonal integration. Note: (a) belowground biomass of older ramets, (b) total biomass of older ramets, and (c) biomass of the entire clonal fragment. Numbers above boxes give probability that means did not differ between parasitism or nutrient treatments.

**Fig. S3.** Ratio of *Cuscuta* biomass to total biomass of the parasitic system. Note: Total biomass of the parasitic system includes both *Cuscuta* and host plant biomass (under spacer‑connected condition). WCp and WTp denote the host plants *Sphagneticola chinensis* and *S. trilobata* in previous studies, respectively, while WTn denotes *S. trilobata* in the present study. Different uppercase letters indicate highly significant differences between groups (*P*<0.01), whereas the same uppercase letters indicate no significant differences (*P*>0.05).
